# Tumor-associated macrophages promote pancreatic ductal adenocarcinoma progression by inducing epithelial-to-mesenchymal transition

**DOI:** 10.18632/aging.202264

**Published:** 2021-01-10

**Authors:** Cheng Xiong, Youwei Zhu, Meilin Xue, Yongsheng Jiang, Yiming Zhong, Lingxi Jiang, Minmin Shi, Hao Chen

**Affiliations:** 1 Department of General Surgery, Pancreatic Disease Center, Ruijin Hospital, Shanghai Jiaotong University School of Medicine, Shanghai, China; 2 Research Institute of Pancreatic Diseases, Shanghai Jiaotong University School of Medicine, Shanghai, China; 3 State Key Laboratory of Oncogenes and Related Genes, Shanghai, China; 4 Institute of Translational Medicine, Shanghai Jiaotong University, Shanghai, China

**Keywords:** pancreatic ductal adenocarcinoma, tumor-associated macrophages, TGF-β signaling pathway, epithelial-mesenchymal transition, metastasis

## Abstract

In this study, we investigated the role of tumor-associated macrophages (TAMs) in the progression of pancreatic ductal adenocarcinoma (PDAC). PDAC patients with higher levels of CD68^+^ TAMs exhibited shorter overall survival. In Transwell assays, PDAC cells incubated with TAMs or conditioned media from TAM cells (TAM-CM) showed higher migration and invasion rates than controls. PET/CT scan analysis of orthotopic PDAC model mice revealed greater primary tumor growth and liver metastasis in the TAM-CM treatment group than the controls. H&E staining of liver tissues showed significantly higher numbers of metastatic nodules in the TAM-CM treatment group. Heat inactivation of TAM-CM significantly reduced Transwell migration by PDAC cells, suggesting the involvement of one or more secreted proteins in PDAC progression. Transcriptome sequencing analysis of PDAC cells treated with TAM-CM revealed significant enrichment of transforming growth factor-β (TGF-β) signaling pathway genes. Western blot and qRT-PCR analysis showed that TAM-CM enhanced PDAC migration cells by inducing epithelial-to-mesenchymal transition through the TGF-β-Smad2/3/4-Snail signaling axis. The pro-tumorigenic effects of TAMs or TAM-CM were abolished by TGF-β signaling pathway inhibitors and neutralizing TGF-β antibody. These results demonstrate that TAMs promote PDAC progression through the TGF-β signaling pathway.

## INTRODUCTION

Pancreatic ductal adenocarcinoma (PDAC) is the seventh leading cause of cancer-related deaths worldwide and develops from the epithelial cells of the pancreatic duct [1, 2]. Nearly 80% of PDAC patients are diagnosed in advanced stages and are not eligible for surgical resection. Hence, the 5-year survival rate of PDAC is less than 5% and the median survival rate is only about 3-6 months [3, 4]. The poor prognosis of PDAC patients is also due to high rates of metastasis rate and chemotherapy resistance [5, 6]. Therefore, it’s important to identify mechanisms underlying metastasis and chemotherapy resistance in PDAC.

Tumor microenvironment (TME) plays an important role in tumor development, progression, response to therapy, and recurrence [7]. In PDAC tissues, TME consists of pancreatic cancer cells, extracellular matrix (ECM), and stromal cells. Stromal cells include pancreatic stellate cells (PSCs), regulatory T cells, myeloid-derived suppressor cells (MDSCs), and tumor-associated macrophages (TAMs), which secrete cytokines and modulate the TME [8]. TAMs are one of the most abundant immune cells and constitute 11% of the total cellular composition in the PDAC tissues [9]. TAMs promote tumor progression by inhibiting immune surveillance and enhance angiogenesis [10]. However, the contribution of TAMs in PDAC progression is still not clear.

Macrophages are classified broadly into M1 and M2 subtypes. M1 macrophages are characterized by high expression of iNOS, whereas, M2 macrophages are characterized by high expression of CD204, CD206, ARG1 and CD163 [11–13]. Furthermore, M1 and M2 macrophages perform diverse functions in tumor progression. M1 macrophages are pro-inflammatory and suppress tumor growth and progression, whereas, M2 macrophages promote tumor progression by antagonizing anti-tumor T-cell immunity [14–18]. Evidence suggests that PDAC cells under hypoxia promote M2 polarization of TAMs, thereby enhancing the metastatic potential of PDAC cells [19]. In gastric cancer, exosomes derived from M2 macrophages contain metastasis-promoting proteins and signaling molecules [20]. M2 macrophages also can promote tumor progression via secreting proteins [16].

In this study, we found that TAMs are enriched in PDAC tissues, and its enrichment showed negative correlation with PDAC overall survival rate. *In vitro* and *in vivo*, TAMs could promote PDAC metastasis through EMT. Furthermore, TGF-β secreted by TAMs rather than exosomes, accelerates PDAC progression via regulating Smand2/3/4-Snail-E-cad signaling axis in PDAC cells. Our study shed a light on novel therapeutic strategies which aim to eliminate the pro-tumoral activity of TAMs.

## RESULTS

### High TAM density correlates with poor overall survival of PDAC patients

**Figure 1:**
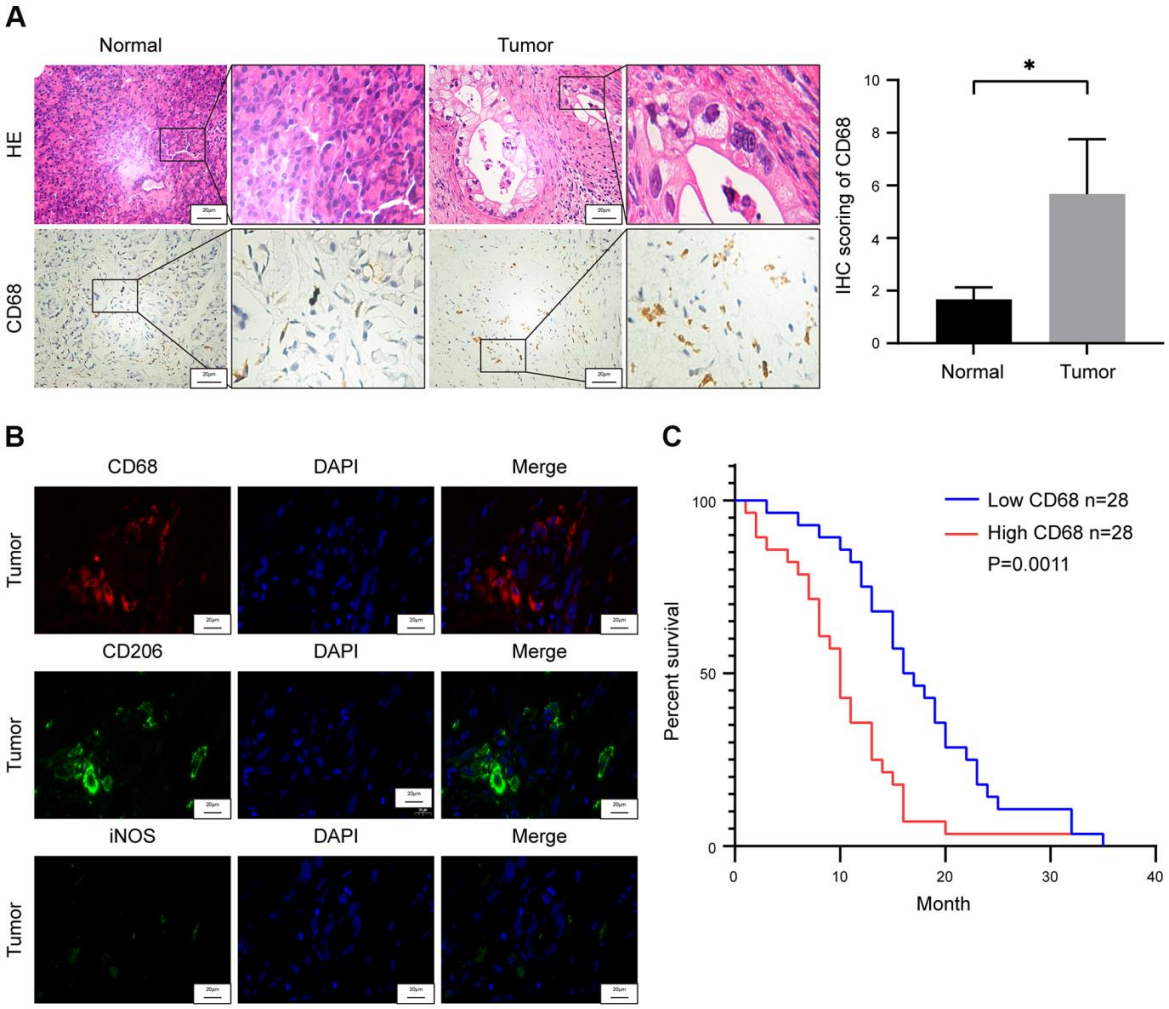
**Tumor-associated macrophage (TAM) density correlates with survival outcomes in pancreatic ductal adenocarcinoma (PDAC) patients.** (**A**) Representative images show hematoxylin and eosin (H&E) and CD68 stained PDAC and adjacent normal pancreatic tissues. Note: n = 3; Scale bar = 20 μm; *P < 0.05. (**B**) Representative fluorescence staining images show CD68, iNOS and CD206 expression in PDAC and adjacent normal pancreatic tissues. Scale bar = 20 μm. (**C**) Kaplan-Meier survival curve analysis shows overall survival rates of CD68-high (n = 28) and CD68-low (n = 28) expressing PDAC patients from Ruijin Hospital (log-rank test: P < 0.01).

We evaluated the proportions of CD68^+^ TAMs in the PDAC tumor and adjacent normal pancreatic tissues (n=3) to determine TAM infiltration levels in human PDAC tissues and their correlation with survival outcomes. Immunohistochemical staining analysis showed significant enrichment of CD68^+^ TAMs in the PDAC tissues compared to the adjacent normal pancreatic tissues (Figure 1A). Macrophages are categorized into M1 and M2 subtypes that exhibit diverse functions in relation to tumor progression [21]. M2-type macrophages are immunosuppressive and are characterized by high expression of CD206, CD204 and ARG1, whereas, M1-type macrophages are pro-inflammatory and exhibit high expression of CD80, iNOS, and CD86 [19]. Most of the CD68^+^ macrophages in PDAC tissues were CD206 positive and showed weak iNOS expression (Figure 1B and Supplementary Figure 1). These results suggest that majority of the TAMs in the human PDAC samples belonged to immunosuppressive M2-phenotype.

We then assessed the association between TAM density and overall survival (OS) rate of patients with PDAC. Based on the IHC staining of CD68 in the paraffin embedded specimens of 56 PDAC patients, we assigned a histochemistry score (H-score) that denotes the infiltration status of TAMs. The PDAC patients were then subdivided into high (H-score >44) and low (H-score <44) TAM infiltration groups based on the median H-score of 44, and compared their OS rates. Kaplan-Meier survival curve analysis showed that the OS rate of PDAC patients with high density of TAMs was significantly lower than the PDCA patients with low density of TAMs (Figure 1C).

### TAMs promote *in vitro* PDAC migration through EMT

**Figure 2:**
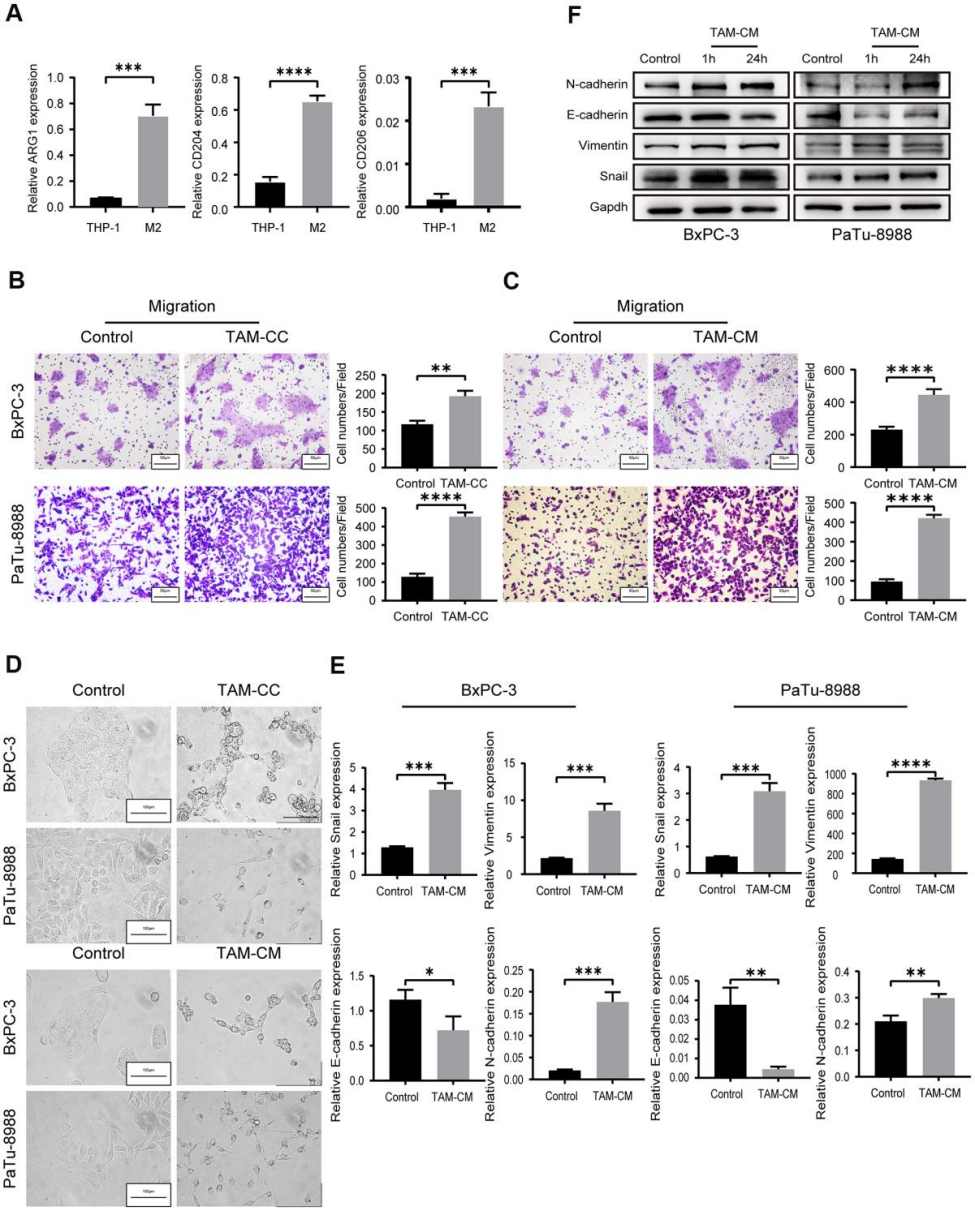
**TAMs promote *in vitro* PDAC cell migration via EMT.** (**A**) QRT-PCR analysis shows relative expression of M2-type macrophage markers such as CD206, CD204, and ARG1 in IL-4-treated THP-1 cells compared to the corresponding controls. ***P < 0.001; ****P < 0.0001. (**B**) Transwell assay results show the migration and invasiveness of BxPC-3 and PaTu-8988 cells co-cultured with TAMs (TAM-CC) and their corresponding controls. Scale bar = 50 μm; **P < 0.01; ****P < 0.0001. (**C**) Transwell assay results show the migration and invasiveness of BxPC-3 and PaTu-8988 cells treated with TAM conditional medium (TAM-CM) and their corresponding controls. Scale bar = 50 μm; ****P < 0.0001. (**D**) Representative images show the morphology of BxPC-3 and PaTu-8988 cells co-cultured with TAMs or TAM-CM and with their corresponding controls. Scale bar = 100 μm. (**E**) QRT-PCR analysis shows the relative mRNA levels of EMT markers, E-cadherin, N-cadherin, and Vimentin in BxPC-3 and PaTu-8988 cells treated with TAM-CM and their corresponding controls. *P < 0.05; **P < 0.01; ***P < 0.001; ****P < 0.0001. (**F**) Western blot analyses show the levels of EMT marker proteins, E-cadherin, N-cadherin, and Vimentin in BxPC-3 and PaTu-8988 cells treated with TAM-CM and their corresponding controls.

We used IL-4 treatment to induce differentiation of the THP-1 monocytes into M2-type macrophages. QRT-PCR analysis showed elevated expression of M2-type macrophage markers, such as, mannose receptor CD206, scavenger receptor CD204, and ARG1 in IL-4 induced THP-1 cells (M2-type macrophages) (Figure 2A).

Next, we analyzed the effects of TAMs on *in vitro* migration and invasion of PDAC cell lines. Therefore, we co-cultured the M2-type macrophages with PDAC cell lines, BxPC-3 and PaTu-8988 cells in Transwell assays and observed significantly higher rate of migration and invasion compared to BxPC-3 and PaTu-8988 alone (Figure 2B and Supplementary Figure 2A). We also harvested supernatants from the M2-type macrophages (TAM-CM) after 24 h starvation. Incubation of BxPC-3 and PaTu-8988 cells with TAM-CM increased their migration and invasion ability compared to the BxPC-3 and PaTu-8988 cells cultured with normal medium without TAM-CM (Figure 2C and Supplementary Figure 2B). Transwell migration rates of BxPC-3 and PaTu-8988 cells in the control group (cultured with normal medium without TAM-CM) were comparable to those co-cultured with THP-1 cells (Supplementary Figure 2C).

Previous evidence suggests that epithelial to mesenchymal transition (EMT) is a critical event that confers migration and invasion abilities to the tumor cells [22]. We observed EMT-related morphological changes which were more elongated and spindle-shaped in the BxPC-3 and PaTu-8988 cells when co-cultured with M2-type macrophages or cultured with TAM-CM-containing medium (Figure 2D). We then analyzed the expression of EMT markers in BxPC-3 and PaTu-8988 cells that were induced for 1 h or 24 h with TAM-CM. QRT-PCR and western blot analysis showed reduced mRNA and protein expression of E-cadherin, and increased mRNA and protein expression of N-cadherin and Vimentin in BxPC-3 and PaTu-8988 cells induced with TAM-CM (Figure 2E, 2F). Moreover, the mRNA and protein expression of Snail, a repressor of E-cadherin, was significantly upregulated in the BxPC-3 and PaTu-8988 cells induced with TAM-CM (Figure 2E, 2F). These results show that M2-type TAMs promote *in vitro* migration of PDAC cell lines by inducing EMT.

### TAMs promote *in vivo* liver metastasis of PDAC cells

**Figure 3:**
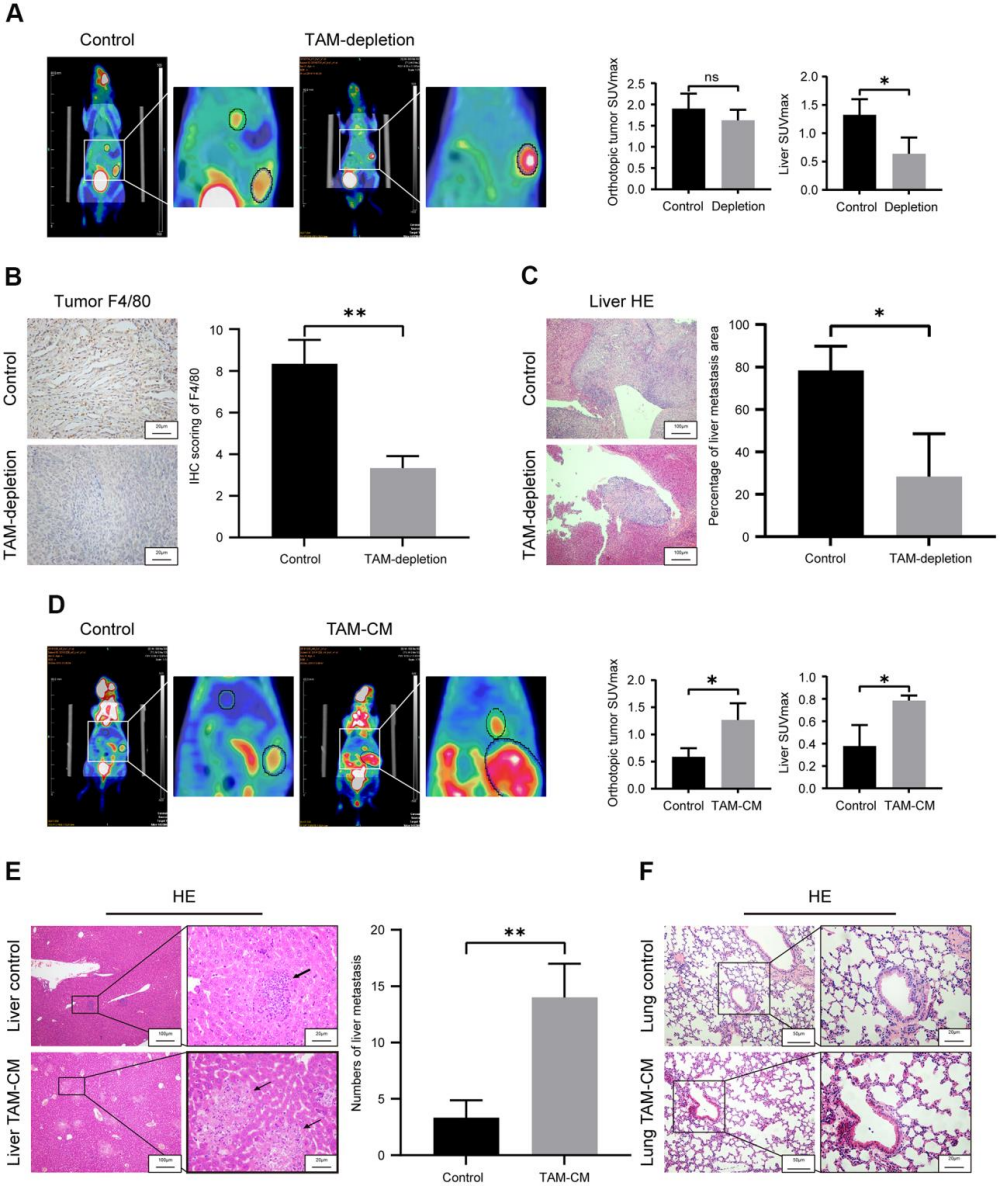
**TAMs promote *in vivo* liver metastasis in the orthotopic PDAC tumor model mice.** (**A**) Positron emission tomography/computed tomography (PET/CT) scanning images show primary pancreatic tumor and liver metastatic lesions in control and TAM-depletion group mice treated with clodronate-liposomes (CLDs). Note: n=6, *P < 0.05. (**B**) IHC staining analysis shows expression of F4/80 in the primary PDAC tumor tissues from the control and TAM-depletion group mice. Scale bar = 20 μm; **P < 0.01. (**C**) Representative images show H&E staining analyses of metastatic lesions in the liver sections from control and TAM-depletion group mice treated with clodronate-liposomes (CLDs). Scale bar = 100 μm, *P < 0.05. (**D**) PET/CT scanning images show primary PDAC tumor and liver metastatic lesions in the control and TAM-CM treatment group mice. Note: n = 6; *P < 0.05. (**E**) Representative images show H&E staining analyses of metastatic lesions in the liver tissues from control and TAM-CM treatment group mice. Scale bar = 20 μm; **P < 0.01). (**F**) Representative images show H&E staining analyses of lung tissues from the control and TAM-CM group mice. Scale bar = 50 μm.

We then assessed the *in vivo* role of TAMs in PDAC metastasis. PET/CT scan analysis of TAM-depleted orthotopic PDAC model mice showed significantly higher liver metastasis in the control group compared to the TAM-depletion group (Figure 3A). TAM-depletion was confirmed by IHC staining with macrophage-specific anti-F4/80 antibody. Primary PDAC tissues of the TAM-depletion group showed significant depletion of TAMs compared to those from the control group (Figure 3B). H&E staining showed that liver metastasis significantly increased in the control group compared to the TAM-depletion group (Figure 3C).

To further confirm the involvement of TAM in PDAC progression, we performed PET/CT scan analysis of control and TAM-CM treated orthotopic PDAC tumor model nude mice at 3 weeks to determine the status of primary tumor and liver metastasis. PET/CT scan results demonstrated that TAM-CM treatment enhanced primary PDAC tumor growth and liver metastasis compared to the control group (Figure 3D). H&E staining showed that the number of liver metastasis nodules were significantly higher in the TAM-CM treatment group compared to the control group (Figure 3E). However, H&E staining did not show any lung metastasis in both TAM-CM and control group mice (Figure 3F). Taken together, these results confirm that TAMs promote *in vivo* PDAC metastasis.

### TAMs activated TGF-β signaling pathway in PDAC cell lines through secreting cytokines

**Figure 4:**
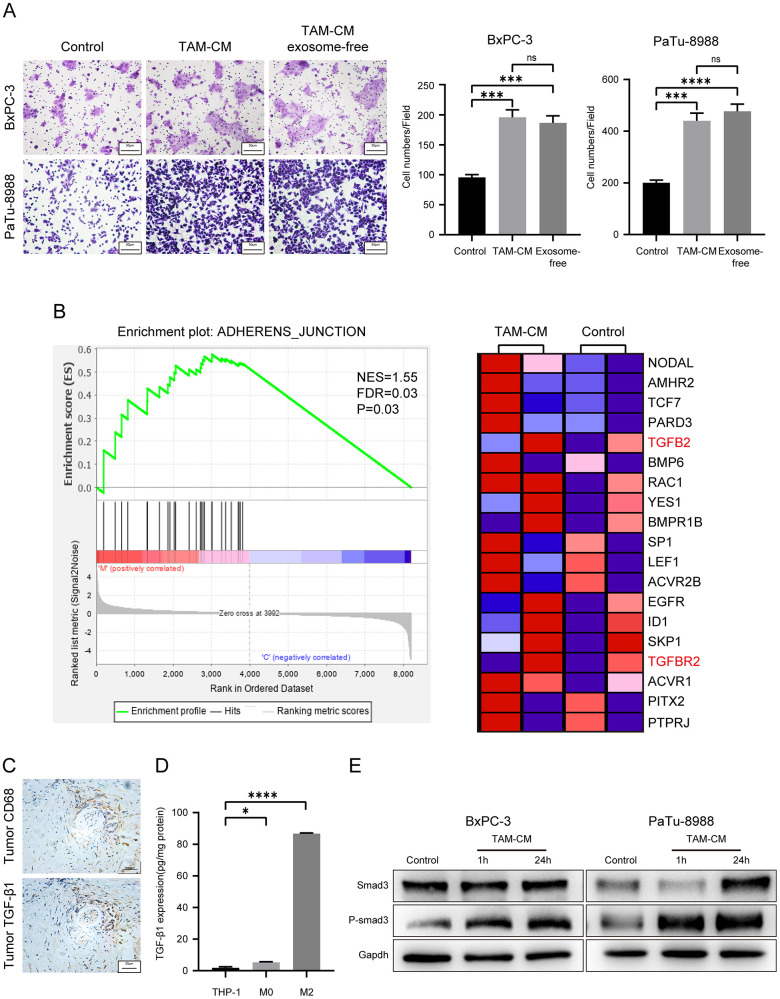
**TAM-derived cytokines activate TGF-β signaling pathway in the PDAC cell lines.** (**A**) Transwell assay results show the migration and invasiveness of BxPC-3 and PaTu-8988 cells incubated with exosome-free TAM-CM or normal TAM-CM. Scale bar = 50 μm; ***P < 0.001; ****P < 0.0001. (**B**) The enrichment plot shows significantly differentially expressed genes in PDAC cells treated with TAM-CM according to the Gene Set Enrichment Analysis (GSEA). (**C**) Representative images show IHC analysis of CD68 and TGF-β staining in the PDAC tissue sections. Scale bar = 20 μm. (**D**) ELISA analysis shows the levels of TGF-β secreted by THP-1, M0-type macrophages and M2-type macrophages. *P < 0.05; ****P < 0.0001. (**E**) Western blot analysis shows the levels of Smad3 and phospho-Smad3 proteins in control and TAM-CM treated BxPC-3 and PaTu-8988 cells.

Next, we analyzed the mechanisms through which M2-type macrophages or TAM-CM enhances the migration ability of the PDAC cell lines. We hypothesized that TAMs may secrete tumor metastasis-promoting exosomes or cytokines. We first investigated the role of TAM-related exosomes in PDAC metastasis *in vitro*. We isolated exosomes from TAM-CM through ultracentrifugation. Western blot analysis showed significant reduction of exosomal markers CD9 in the exosome-depleted TAM-CM compared to the normal TAM-CM (Supplementary Figure 3A). Transwell migration assays showed that exosome-free TAM-CM enhanced the migration ability of BxPC-3 and PaTu-8988 cells comparable to the control TAM-CM group (Figure 4A). This suggested that the TAM-derived exosomes did not play a significant role in promoting *in vitro* PDAC metastasis.

Next, we tested if the secretory proteins in the TAM-CM increase the migration ability of PDAC cells. Transwell assay results showed that the migration ability of BxPC-3 and PaTu-8988 cells incubated with heat-inactivated TAM-CM was significantly reduced compared to those incubated with normal control medium (Supplementary Figure 3B). These results demonstrate that secretory proteins derived from TAMs promote PDAC progression.

We then compared transcriptome profiles of the BxPC-3 and PaTu-8988 cells treated with TAM-CM or normal medium and identified 121 differentially expressed genes between the control and TAM-CM treatment groups (Supplementary Figure 3C). Gene set enrichment analysis (GSEA) of the differentially expressed genes showed enrichment of the “ADHERENS_JUNCTION” gene set including TGF-β and TGF-β receptors (Figure 4B). IHC analysis of PDAC tissue sections showed co-localization of CD68 and TGF-β1 (Figure 4C). ELISA assay showed that M2-type macrophages secreted significantly higher levels of TGF-β1 than the THP-1 and M0-type macrophages (Figure 4D). ELISA assays also showed that TAM-CM didn’t induce secretion of TGF-β1 from the PDAC cells (Supplementary Figure 3D). Western blot analysis showed significantly higher levels of phosphorylated Smad3 in BxPC-3 and PaTu-8988 cells incubated with TAM-CM compared to those incubated with normal medium without TAM-CM (Figure 4E). These data demonstrate that TAMs promote PDAC metastasis by secreting TGF-β and activating the TGF-β signaling pathway in PDAC cells.

### Inhibition of TGF-β signaling pathway attenuates EMT in the PDAC cell lines

**Figure 5:**
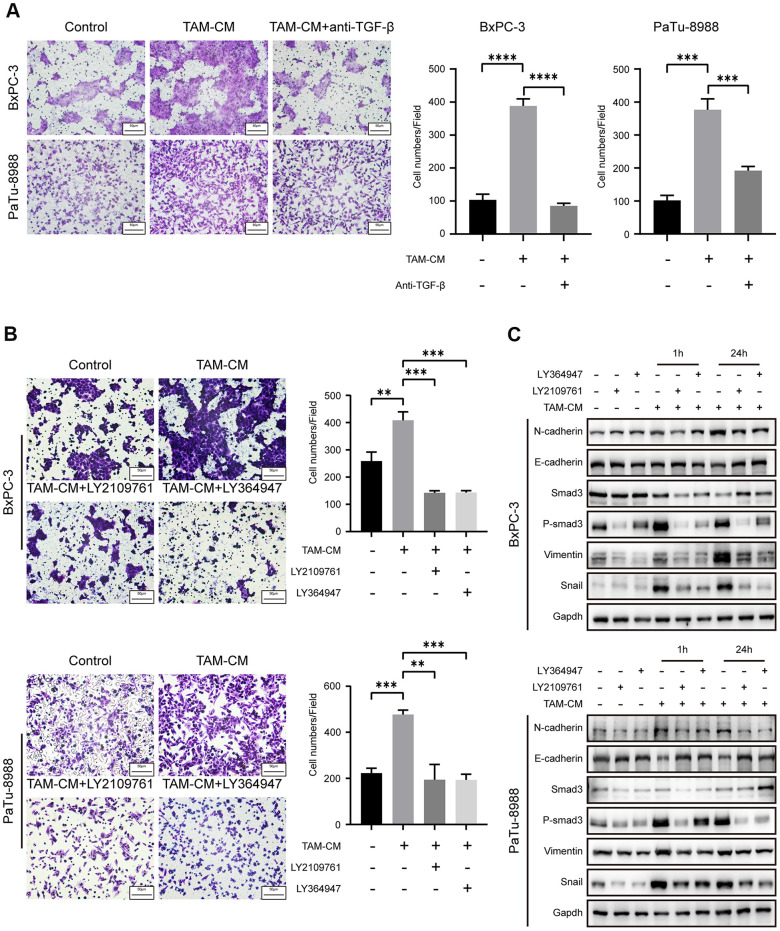
**Inhibition of TGF-β signaling pathway attenuates EMT of PDAC cell lines.** (**A**) Transwell assay results show migration and invasion ability of BxPC-3 and PaTu-8988 cells after incubation with TAM-CM in the presence or absence of the neutralizing TGF-β neutralizing antibody. Scale bar = 50 μm; ***P < 0.001; ****P < 0.0001. (**B**) Transwell assay results show the migration and invasion ability of BxPC-3 and PaTu-8988 cells after incubation with TAM-CM in the presence or absence of TGF-β receptor inhibitors, LY364947 (1 μM) and LY2109761 (10 μM). Scale bar = 50 μm; **P < 0.01; ***P < 0.001. (**C**) Western blot analysis shows the expression of EMT markers (E-cadherin, N-cadherin, and Vimentin), Smad3 and phospho-Smad3 proteins in BxPC-3 and PaTu-8988 cells incubated with TAM-CM in the presence or absence of TGF-β receptor inhibitors, LY364947 and LY2109761.

Next, we verified if activation of TGF-β signaling pathway enhanced migration ability of the PDAC cell lines. Transwell assays showed that migration and invasion of PDAC cell lines treated with TAM-CM and neutralizing anti-TGF-β antibody was significantly reduced compared to the PDAC cells treated with TAM-CM alone (Figure 5A and Supplementary Figure 4). Moreover, PDAC cell lines treated with TGF-β receptor inhibitors, LY2109761 and LY364947, in presence of TAM-CM showed decreased Transwell migration rates compared to BxPC-3 and PaTu-8988 cells treated with TAM-CM alone (Figure 5B). Previous studies demonstrate that the phospho-Smad2/3/4 complex promotes transcription of Snail and Zeb family members [23]. Western blot analyses showed that the TGF-β receptor inhibitors decreased the phosphorylation of Smad3 and Snail after TAM-CM treatment (Figure 5C). Furthermore, TGF-β receptor inhibition in TAM-CM treated PDAC cells decreased the levels of mesenchymal markers, Vimentin and N-cadherin, and increased the levels of epithelial marker, E-cadherin (Figure 5C). Taken together, these results demonstrate that inhibition of TGF-β signaling pathway attenuates induction of EMT in TAM-CM treated PDAC cells.

## DISCUSSION

Pancreatic ductal adenocarcinoma is the seventh leading cause of cancer-related deaths and is associated with high recurrence rates [24]. PDAC is also a highly chemo-resistant tumor with abundant fibrotic mass, which adversely affects drug delivery [19]. Moreover, previous studies have demonstrated that the tumor microenvironment (TME) plays an important role in PDAC progression [25]. Pancreatic tumor microenvironment consists of fibroblasts, extracellular matrix, endothelial cells and immune cells. TAMs are the most abundant immune cells in the PDAC TME and constitute 11% of the total stromal cells in the PDAC tissues [9]. Macrophages are divided into two subtypes: M1-type macrophages, which are pro-inflammatory and anti-tumorigenic, and M2-type macrophages, which are immunosuppressive and promote tumor progression [26].

TAMs enhance tumor cell proliferation, migration, genetic instability, angiogenesis and tissue remodeling [27]. The correlation between TAMs and cancer prognosis can vary between different solid cancers. For example, TAM density is positively associated with advanced stage breast and bladder cancers [28, 29], but, this relationship is reversed in ovarian and gastric cancers [30, 31]. The mechanistic role of TAMs in PDAC is still unclear.

**Figure 6:**
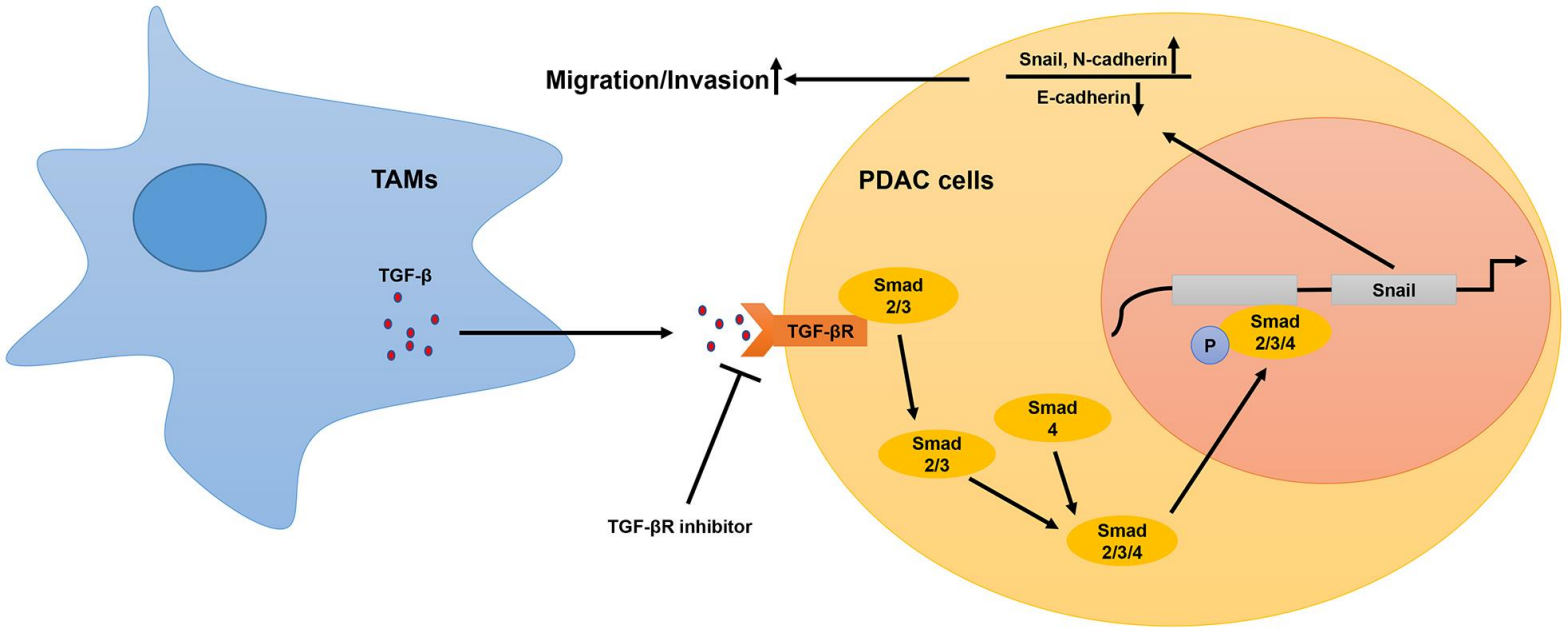
**Schematic representation of the mechanistic role of TAMs in PDAC progression.** In the tumor microenvironment, TAMs secrete TGF-β, which binds to the TGF-β receptors on the surface of PDAC cells and activates the TGF-β signaling pathway. This includes translocation of phosphorylated Smad2/3 (transcription factor) into the nucleus from the cytoplasm. The phospho-Smad2/3/4 complex upregulates Snail, a key transcriptional factor required for the expression of EMT-related genes. Subsequently, EMT promotes metastasis of PDAC cells.

Our study demonstrates that high infiltration of TAMs in PDAC tissues is associated with poor overall survival (OS). Moreover, we demonstrated that M2-like TAMs promote *in vitro* and *in vivo* PDAC cell migration and invasion. This indicated that TAMs promoted PDAC metastasis. Previous studies have reported that TAMs promote PDAC progression through exosomes [20]. However, our study shows that TAM-derived secretory cytokines such as TGF-β promote PDAC cell migration. We also show that TAM-related exosomes do not play a significant role in PDAC progression. Transcriptional profiling showed that TAMs activate the TGF-β signaling pathway in the PDAC cells. Previous studies demonstrate that M2-type macrophages secrete higher levels of TGF-β than M1 macrophages and other macrophage subtypes [32]. Our study demonstrates that TGF-β binds to the TGF-β receptors on the PDAC cell surface and triggers phosphorylation of Smad2/3, which eventually forms the active phospho-Smad2/3/4 complex that promotes transcription of Snail. Snail decreases the expression of E-cadherin and promotes PDAC metastasis. Our study demonstrates that TGF-β receptor inhibitors abrogate the tumor-promoting effects of TAMs. (Figure 6).

The five strategies currently used to block TGF-β signaling pathway in human cancer include blocking antibodies and ligand traps, antisense oligos, TβRII/ALK5 inhibitors, immune response-based strategies, and other inhibitors of the TGF-β pathway. Several compounds that block TGF-β signaling have shown encouraging results in the mouse PDAC models, and GC1008 (Fresolimumab) is an antibody against TGF-β, which has successfully completed phase I trial in renal cell carcinoma and advanced melanoma patients (NCT00356460) [33]. The results of our study suggest that therapeutic strategies targeting TGF-β pathway may be beneficial for PDAC patients.

Tumor microenvironment is a complex setting involving dynamic interactions between several different cell types and signaling molecules. The proteins secreted by the TAMs can modulate the functions and biological behavior of tumor cells and other stromal cells. For example, studies have shown that TAMs diminish the killing of tumor cells by cytotoxic T cells and NK cells [34]. Moreover, TAMs promote fibrosis and ECM remodeling by activating fibroblasts to secrete ECM-related proteins and other matrix components [35]. Afik et al showed that TAMs promote colon cancer development by remodeling the extracellular matrix composition and structure [36]. Peng et al used single-cell RNA-seq analysis to identify five different clusters of macrophages in PDAC tumor tissues, all of which have different characteristics and roles; for example, cluster 2 TAMs express genes related to extracellular matrix deposition and remodeling, whereas, cluster 3 TAMs express genes related to the migration of myeloid-derived suppressor cells [9]. Furthermore, macrophages of different origins have differential effects on tumor progression. Monocyte-derived macrophages show limited effects on tumor progression, whereas, tissue-resident macrophages promote tumor progression [37]. Our study explored TAM-related mechanisms that promote PDAC metastasis. However, further investigations are necessary to confirm our results and to identify the roles of other macrophage subpopulations in PDAC progression in order to design effective PDAC immunotherapies.

In conclusion, our study demonstrates that TAMs in the PDAC microenvironment promote metastasis by secreting TGF-β, which activates the downstream phospho-Smad2/3/4-Snail axis in PDAC cells. Snail upregulation induces EMT of the PDAC cells, thereby enhancing their metastatic and invasive properties. We also demonstrate that TGF-β signaling pathway inhibition abrogates enhanced migration ability of PDAC cells. Therefore, our study demonstrates that neutralizing TGF-β antibodies may be effective in improving survival outcomes of PDAC patients.

## MATERIALS AND METHODS

### Patient specimens and ethical statement

We obtained PDAC tumor and adjacent normal tissue specimens from 56 PDAC patients that underwent surgery between 2012 and 2018 at the Pancreatic Disease Center of the Ruijin Hospital, Shanghai, China. The study was approved by the Ethics committee of Ruijin Hospital affiliated to the Shanghai Jiaotong University School of Medicine, Shanghai, China and conducted according to the ethical principles adopted by the World Medical Association in the Declaration of Helsinki and local ethical legislations for conducting research involving human subjects. All patients signed informed consent forms.

### Cell lines and cell culture

We obtained the pancreatic cancer cell line, BxPC-3, and the monocytic cell line, THP-1, from American Type Culture Collection. The human pancreatic cancer cell line, PaTu-8988, and the mouse pancreatic cancer cell line, Pan02, were obtained from the Cell Resource Center, Shanghai Institute of Biochemistry and Cell Biology at the Chinese Academy of Sciences, Shanghai, China. All cell lines were cultured in RPMI-1640 medium supplemented with heat-inactivated 10% fetal bovine serum (FBS, Gibco, USA) and 1% penicillin/streptomycin in a standard humidified incubator at 37° C and 5% CO_2_. We verified all cell lines using Short Tandem Repeat (STR) analyses.

### Induction of THP-1 monocytes to M2-polarized TAMs

We treated THP-1 monocytes with 100 ng/ml phorbol 12-myristate 13-acetate (PMA, Sangon Biotech, China) for 48 h followed by 20 ng/ml IL-4 (Peprotech, USA) for 24 h to obtain M2-polarized TAMs. Then, the THP-1 cells were further cultured in serum-free RPMI-1640 medium for 24 h. The culture supernatants were collected, centrifuged at 300 xg for 5 minutes, filtered through a 0.22 μm filter mesh, and stored at -80° C as TAM conditioned medium (TAM-CM).

### Immunohistochemistry

Immunohistochemistry analysis was performed as previously described [38]. Briefly, the formalin-fixed paraffin embedded mouse and human PDAC tissue blocks were subjected to immunohistochemical staining using primary rabbit anti-F4/80 (1:100; Proteintech, USA), rabbit anti-CD68 (1:100; Proteintech, USA) and rabbit anti-TGF-β (1:100; Servicebio, China) antibodies, followed by incubation in secondary biotinylated antibody and finally visualized with DAB solution (Servicebio, China) and counterstained with hematoxylin (Servicebio, China). Picture were taken under a light microscope (Olympus BX50, Japan).

### Immunofluorescence

Samples were blocked in 5% bovine serum albumin in phosphate buffered saline (PBS) for 1 h, rabbit anti-CD68 (1:100; Servicebio, China), rabbit anti-CD206 (1:100; Servicebio, China) and rabbit anti-iNOS (1:100; Servicebio, China) were incubated at 4° C overnight. The secondary antibodies Alexa Fluor 568-conjugated anti-rabbit IgG (1:200; Invitrogen, UK) and Alexa Fluor 488-conjugated anti-rabbit IgG (1:200; Invitrogen, UK) were applied. The nuclear was stained by 4’, 6-diamidino-2-phenylindole (DAPI, Sigma, USA). Images were captured with a fluorescent microscope (Leica TCS SP2).

### Western blotting

Total cellular proteins were extracted with RIPA buffer (Solarbio, China) and Protease and Phosphatase Inhibitor cocktail (1:100; New Cell and Molecular Biotech, China), which was added immediately before cell lysis. The protein amounts were quantified using the BCA assay (Thermo Fisher Scientific, USA). Then, equal amounts of proteins were separated on SDS-PAGE and transferred to PVDF membranes. The membranes were then blocked with 5% skimmed milk in TBST at room temperature for 30 mins. The blots were then incubated overnight at 4° C with primary antibodies: mouse anti-CD9 (1:1000; Santa Cruz, USA), rabbit anti-E-cadherin (1:1000; Proteintech, USA), rabbit anti-Vimentin (1:1000; Proteintech, USA), rabbit anti-Snail (1:1000; Cell Signaling Technology, USA), mouse anti-N-cadherin (1:1000; Proteintech, USA), rabbit anti-Smad3 (1:1000; Cell Signaling Technology, USA) and rabbit anti-phosphor-Smand3 (1:1000; Cell Signaling Technology, USA). Rabbit anti-GAPDH (1:1000; Cell Signaling Technology, USA) and rabbit anti-β-actin (1:1000; Cell Signaling Technology, USA) were used as control. The blots were then incubated with HRP-conjugated polyclonal goat anti-rabbit IgG (1:5000; Proteintech, USA) or HRP-conjugated polyclonal goat anti-mouse IgG (1:5000; Proteintech, USA) at room temperature for 1 h. Then, the blots were developed using ECL detection reagent (Meilun Biological Technology, China). The protein bands were visualized, photographed, and quantified using the Image J software.

### Transwell cell migration and invasion assays

We used 24-well Transwell chambers (Corning, USA) to analyze cellular migration and invasiveness. Transwell chamber inserts were coated with matrigel for the invasion assay. M2-polarized TAMs (5×10^5^ cells/well) in RPMI-1640 medium with 20% FBS, TAM-CM with 20% FBS, or RPMI-1640 medium with 20% FBS were added to the bottom chamber, while PDAC cells (1×10^5^ cells/insert) in serum-free RPMI-1640 medium were seeded in the upper chamber. The Transwell chambers with cells were incubated for 24 h. Then, the cells in the upper chamber were removed with cotton plugs. The cells that migrated to the bottom chamber or invaded the matrigel were stained with 1% crystal violet for 5 minutes, and observed under a light microscope (Olympus BX50, Japan). The stained PDAC cells (migrating and invasive) were imaged and counted in five random fields for each sample.

### Quantitative real-time PCR

Total RNA was isolated using Trizol (Invitrogen, USA) according to the manufacturer’s protocol. Equal amounts of total RNA were reverse transcribed into cDNA (TOYOBO, Japan). Then, quantitative PCR was performed using SYBR Green (TOYOBO, Japan) in a real time PCR machine (Analytik Jena, Germany). The primer sequences used for qPCR are as follows: GAPDH forward primer: 5’-ACAACTTTGGTATCGTGGAAGG-3’; GAPDH reverse primer: 5’-GCCATCACGCCACAGTTTC-3’; Vimentin forward primer: 5’-TTGCCGTTGAAGCTGCTAACTACC-3’; Vimentin reverse primer: 5’-AATCCTGCTCTCCTCGCCTTCC-3’; CD206 forward primer: 5’-GACGTGGCTGTGGATAAATAAC-3’; CD206 reverse primer: 5’-CAGAAGACGCATGTAAAGCTAC-3’; ARG1 forward primer: 5’-GGTTTTTGTTGTTGCGGTGTTC-3’; ARG1 reverse primer: 5’-CTGGGATACTGATGGTGGGATGT-3’; CD204 forward primer: 5’-CAGGAAATTCTGGACCAAAAGG-3’; CD204 reverse primer: 5’-CAGCGATCGTCACAAATTGTAC-3’; Snail forward primer: 5’-CTGTGACAAGGAATATGTGAGC-3’; Snail reverse primer: 5’-CTAATGTGTCCTTGAAGCAACC-3’; E-cadherin forward primer: 5’-AGTCACTGACACCAACGATAAT-3’; E-cadherin reverse primer: 5’-ATCGTTGTTCACTGGATTTGTG-3’; N-cadherin forward primer: 5’-CGATAAGGATCAACCCCATACA-3’; N-cadherin reverse primer: 5’-TTCAAAGTCGATTGGTTTGACC-3’. GAPDH was used as the internal control. Relative gene expression was estimated using the 2^-ΔΔCt^ method.

### Orthotopic PDAC model mice

We treated wild-type C57BL/6 mice with 1 mg clodronate-liposomes (CLDs) (Yeasen, China) via tail vein to deplete macrophages. After a three-day recovery, we established the orthotopic PDAC model mice by injecting 5×10^6^ Pan02 cells into the pancreas of the macrophage-depleted C57BL/6 mice. The TAM-depletion group mice received CLD injections every week, whereas, the control group was not injected with CLDs. We performed PET/CT scan at 2 weeks after PDAC implantation to assess the status of the primary tumor and liver metastasis in both groups of mice. Four weeks after orthotopic PDAC implantation, we euthanized the mice and harvested the primary tumor and liver tissues for further experiments.

To determine the actual role of TAMs in PDAC progression, we treated PaTu-8988 cells with TAM-CM for two days and then generated the orthotopic PDAC tumor model mice by implanting 5×10^6^ TAM-CM treated or untreated PaTu-8988 cells into the pancreas of nude mice. We then intraperitoneally injected 50 μl TAM-CM (20 μg/μl protein) every week into the TAM-CM treatment group mice. Three weeks after implantation, we performed PET/CT scan analysis to determine the status of primary tumor and liver metastasis.

### ELISA

We estimated TGF-β levels in the TAM cell supernatants using the ELISA kit (MultiSciences, China) according to manufacturer’s instructions. The samples were measured in duplicate using a Labsystems Multiskan Plus plate reader (test wavelength: 450 nm; reference wavelength: 570 nm) and analyzed using the DeltaSoft JV software.

### Exosome isolation

The cell lines were cultured in the normal medium until 80%–90% confluent. Thereafter, medium was replaced with RPMI1640 without FBS and cultured for 24 h. Then, the cell culture medium was harvested and centrifuged at 300 g for 10 minutes, 2000 g for 10 minutes, 10000 g for 30 minutes to remove residual cells and debris, and ultracentrifuged at 100000 g for 70 minutes (Beckman Coulter). Then, the exosome-depleted supernatant was stored at -80° C until further use.

### Supernatant concentration

The cell lines were cultured in the normal medium until 80%–90% confluent. Thereafter, medium was replaced with RPMI1640 without FBS and cultured for 24 h. Then, the cell culture medium was harvested and centrifuged at 1200 rpm for 3 minutes to remove residual cells and debris. Then, the supernatant was concentrated using Amicon Ultra-4 centrifugal filter devices (3000 MWCO cutoff) (Millipore) by successive spinning (4 ml of supernatant per spinning) at 5976 rpm for 25 minutes. Protein concentrations were determined by Bradford assay and 50 μg of proteins were used for mass spectrometry sample preparation. The concentrated supernatant was collected and stored at -80° C for further use.

### Statistical analyses

All statistical analyses were performed by SPSS statistics 22.0 software (IBM, USA). Student t-tests were used to compare continuous variables between groups. Chi-square tests were used to examine differences between categorical variables among subgroups. Kaplan-Meier survival curves and log-rank test were used to estimate survival differences. P < 0.05 was considered statistically significant.

## Supplementary Material

Supplementary Figures
