# Supplementary material for: Tumor-associated macrophages promote pancreatic ductal adenocarcinoma progression by inducing epithelial-to-mesenchymal transition
Source: Aging (Albany NY). 2021 Jan 10;13(3):3386–404. doi: 10.18632/aging.202264 (PMC7906203; doi:10.18632/aging.202264)
Supplement: Supplementary Figures [file aging-13-202264-s001.pdf]

SUPPLEMENTARY FIGURES

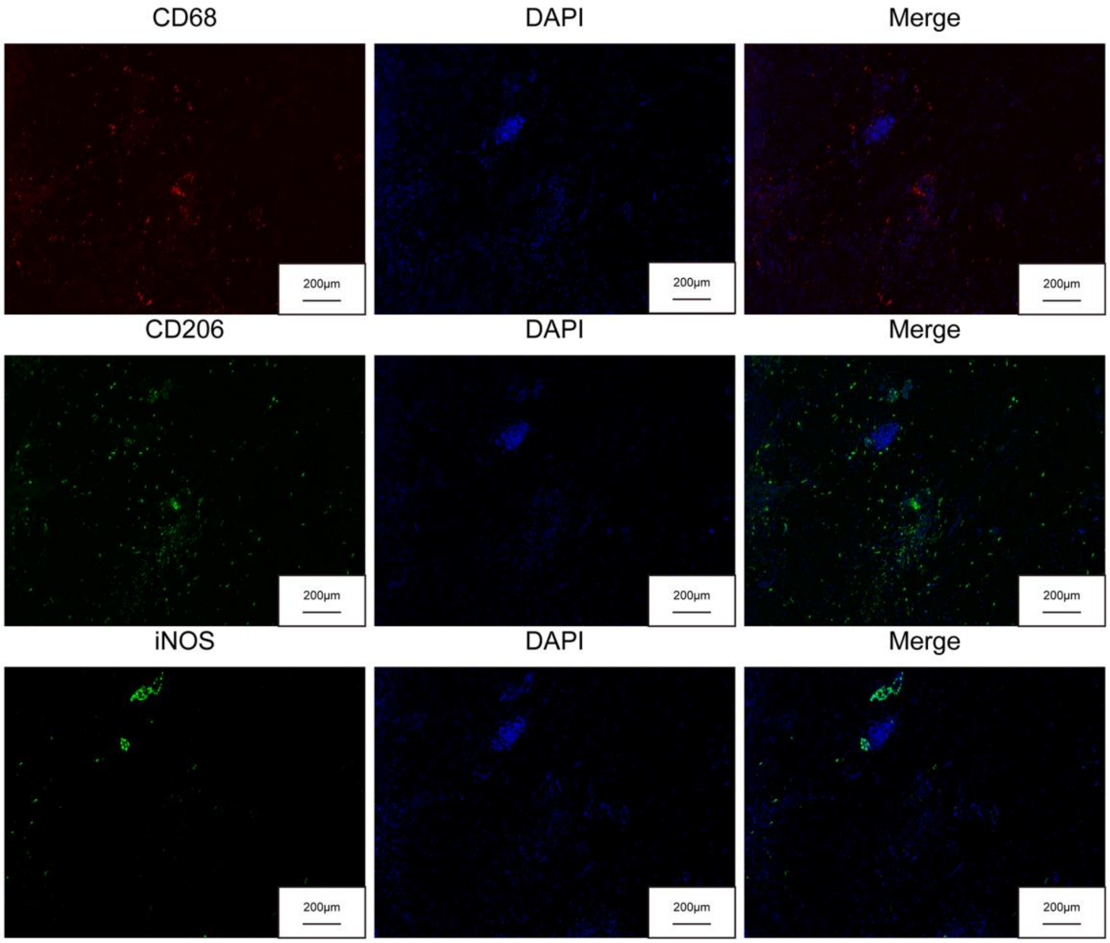

Supplementary Figure 1. Representative fluorescence microscopy images show the staining of CD68, iNOS, and CD206 in the PDAC tissues (Scale bar = 200 μm).

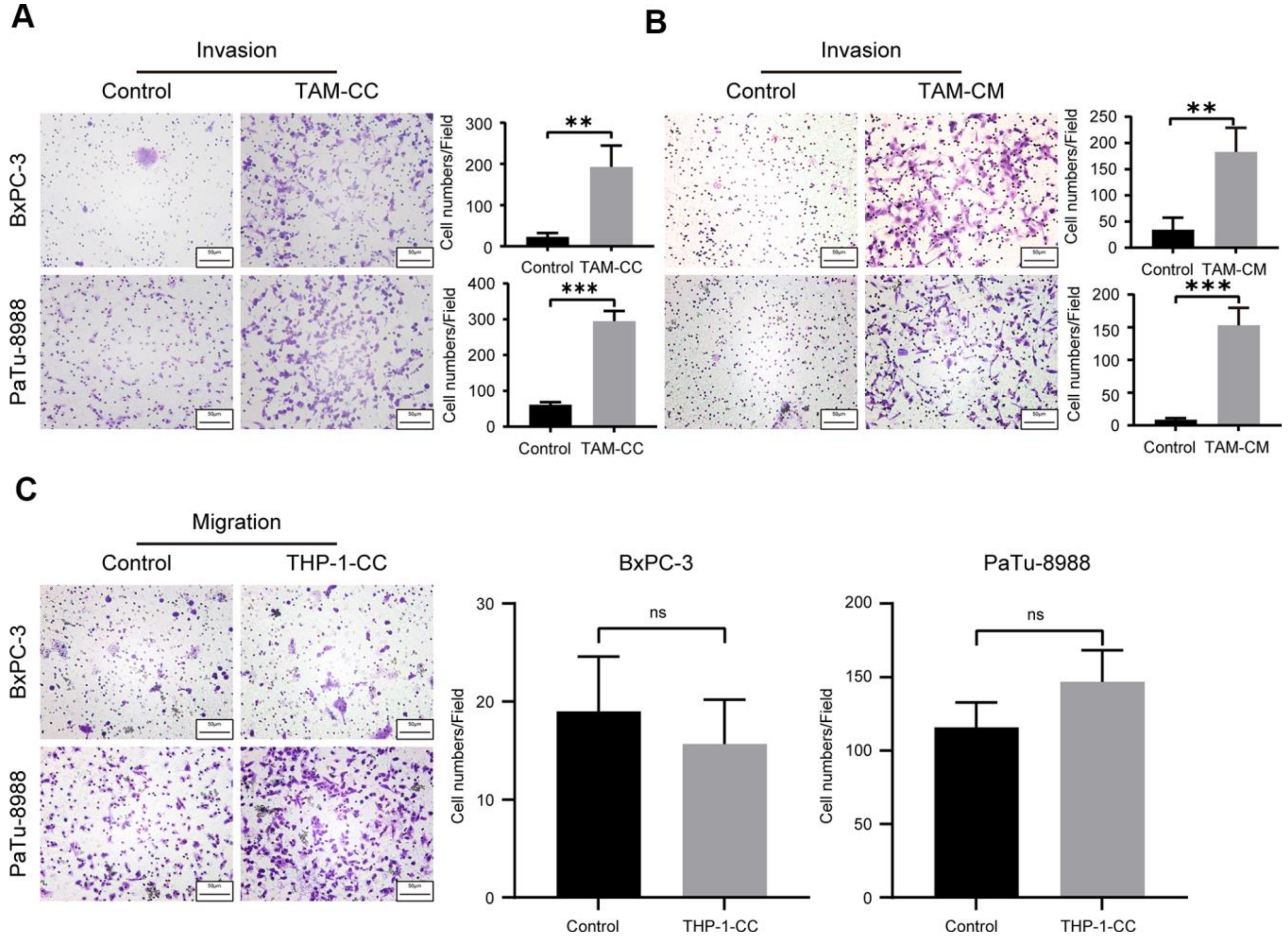

Supplementary Figure 2. (**A**) Transwell assay results show invasiveness of BxPC-3 and PaTu-8988 cells that are co-cultured with TAMs (TAM-CC). Scale bar = 50 µm; **P < 0.01, ***P < 0.001. (**B**) Transwell assay results show invasiveness of BxPC-3 and PaTu-8988 cells after treatment with TAM-CM. Scale bar = 50 µm, **P < 0.01, ***P < 0.001. (**C**) Transwell assay results show migration ability of BxPC-3 and PaTu-8988 cells that are co-cultured with THP-1 cells. Scale bar = 50 µm.

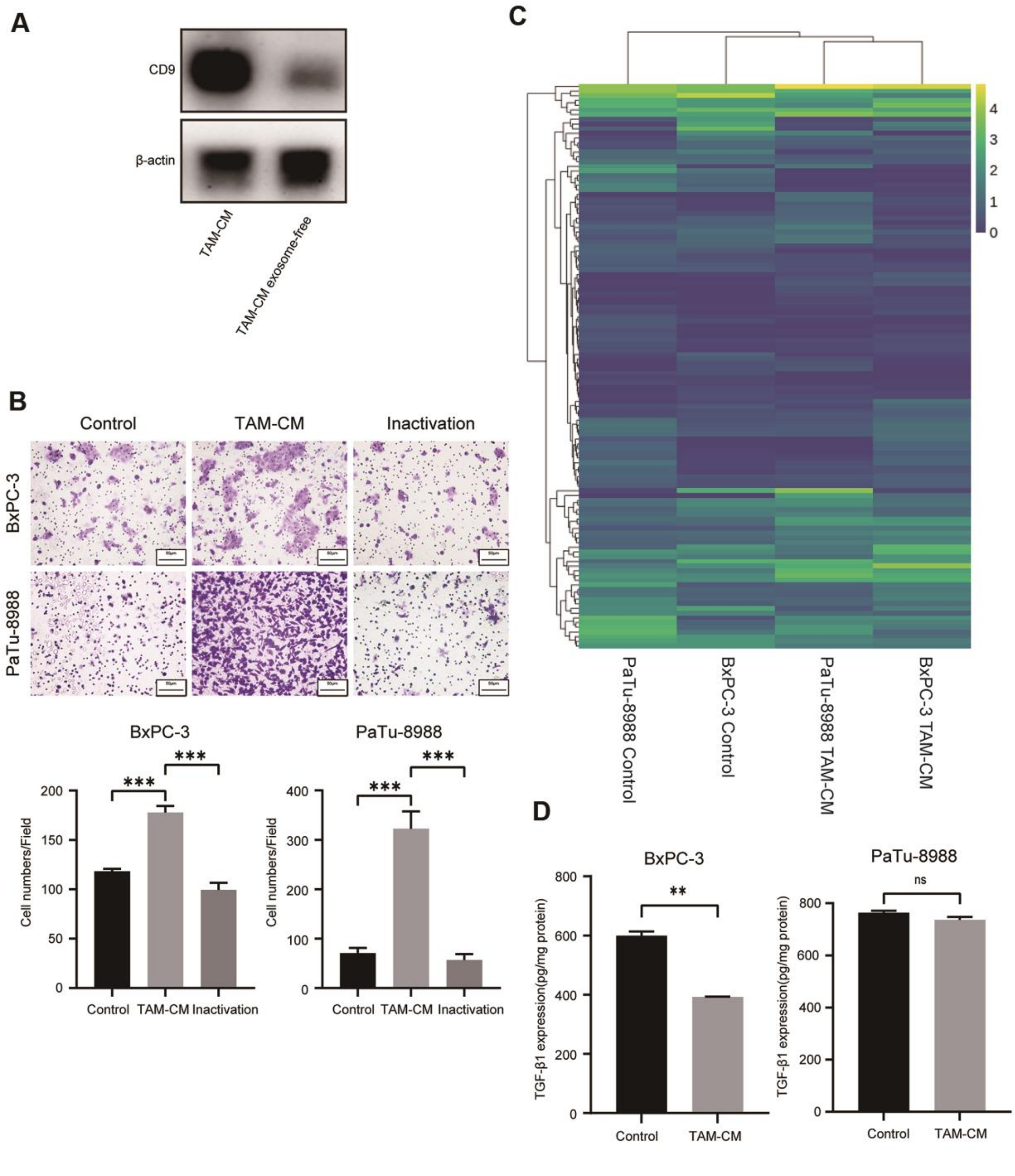

Supplementary Figure 3. (**A**) Western blot analysis shows the levels of exosome marker proteins CD9 in TAM-CM and exosome-free TAM-CM. (**B**) The heatmap shows 121 differentially expressed genes in the TAM-CM treated BxPC-3 and PaTu-8988 cells compared to the corresponding controls. (**C**) Transwell assay results show migration and invasiveness of BxPC-3 and PaTu-8988 cells incubated with normal TAM-CM or inactivated TAM-CM. Scale bar = 50 µm; ***P < 0.001. (**D**) ELISA analysis shows the levels of TGF-β secreted by BxPC-3 or PaTu-8988 cells incubated with or without TAM-CM. **P < 0.01.

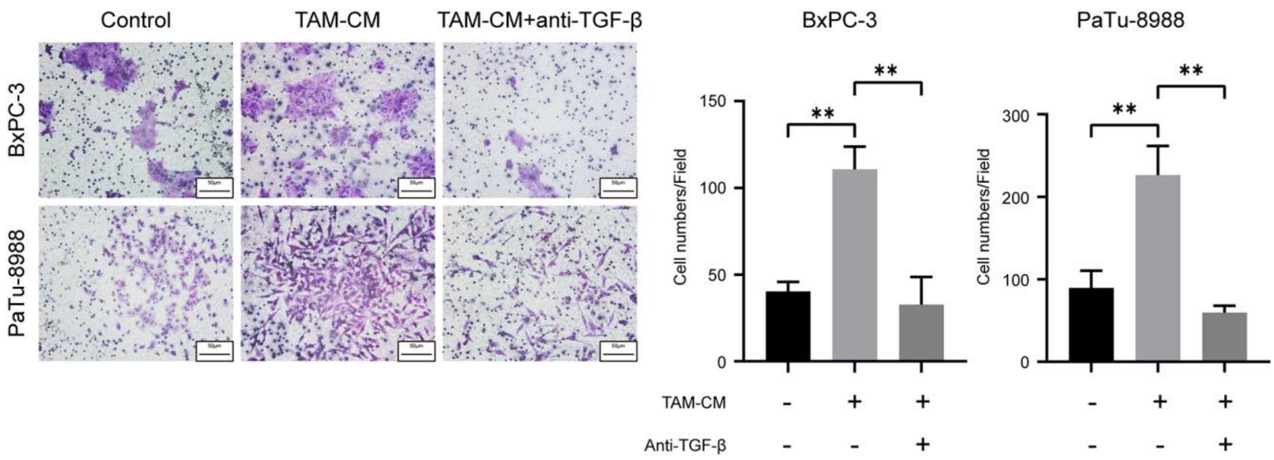

Supplementary Figure 4. Transwell assays show the invasiveness of BxPC-3 and PaTu-8988 cells incubated with TAM-CM in presence or absence of the neutralizing TGF-β antibody. Scale bar = 50 μm; $^{**}P < 0.01$.
